# Author Correction: Spectroscopic evidence for a large spot on the dimming Betelgeuse

**DOI:** 10.1038/s41467-021-25602-7

**Published:** 2021-08-27

**Authors:** Sofya Alexeeva, Gang Zhao, Dong-Yang Gao, Junju Du, Aigen Li, Kai Li, Shaoming Hu

**Affiliations:** 1 CAS Key Laboratory of Optical Astronomy, National Astronomical Observatories, Chinese Academy of Sciences, Beijing, China; 2 School of Astronomy and Space Science, University of Chinese Academy of Sciences, Beijing, China; 3 Shandong Key Laboratory of Optical Astronomy and Solar-Terrestrial Environment, Institute of Space Sciences, School of Space Science and Physics, Shandong University, Weihai, China; 4 School of Astronomy and Space Science, Key Laboratory of Ministry of Education, Nanjing University, Nanjing, China; 5 Department of Physics and Astronomy, University of Missouri, Columbia, MO USA

**Keywords:** Stars, Stellar evolution

Correction to: *Nature Communications* 10.1038/s41467-021-25018-3, published online 5 August 2021.

The original version of this Article contained an error in the author affiliations.

Affiliation 3 incorrectly read Shandong Key Laboratory of Optical Astronomy and Solar-Terrestrial Environment, School of Space Sciences, Shandong University, Weihai, Shandong, 264209, China. The correct affiliation is Shandong Key Laboratory of Optical Astronomy and Solar-Terrestrial Environment, Institute of Space Sciences, School of Space Science and Physics, Shandong University, Weihai, China.

This has now been corrected in both the PDF and HTML versions of the Article.

