# Supplementary material for: Spectroscopic evidence for a large spot on the dimming Betelgeuse
Source: Nat Commun. 2021 Aug 5;12:4719. doi: 10.1038/s41467-021-25018-3 (PMC8342547; doi:10.1038/s41467-021-25018-3)
Supplement: Supplementary file 1 — Supplementary Information [file 41467_2021_25018_MOESM1_ESM.pdf]

Supplementary Information for

Spectroscopic evidence for a large spot on the dimming Betelgeuse

Sofya Alexeeva[1], Gang Zhao[1,2,*], Dong-Yang Gao[3,4], Junju Du[3],
Aigen Li[5], Kai Li[3], and Shaoming Hu[3]
* Corresponding author. E-mail: gzhao@nao.cas.cn

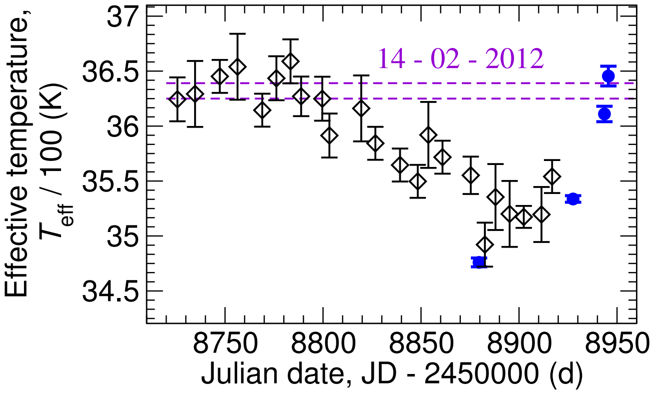

Supplementary Figure 1: **Comparison of our effective temperatures with previous study.** Effective temperatures ($T_{\rm eff}$) derived from the TiO index in supp. ref. 1 (open rhombii) and in this work (filled circles). Error bars for $T_{\rm eff}$ from supp. ref. 1 correspond to standard deviation. Uncertainty for $T_{\rm eff}$ in this work is defined as the difference between the 84th and 50th percentile as the upper limit, and the difference between the 50th and 16th percentile as the lower limit. Two horizontal dashed lines show the ranges $T_{\rm eff}$ for pre-dimming, which we have obtained with ESPaDOnS's spectrum observed on 2012 February, 14. Source data are provided as a Source Data file.

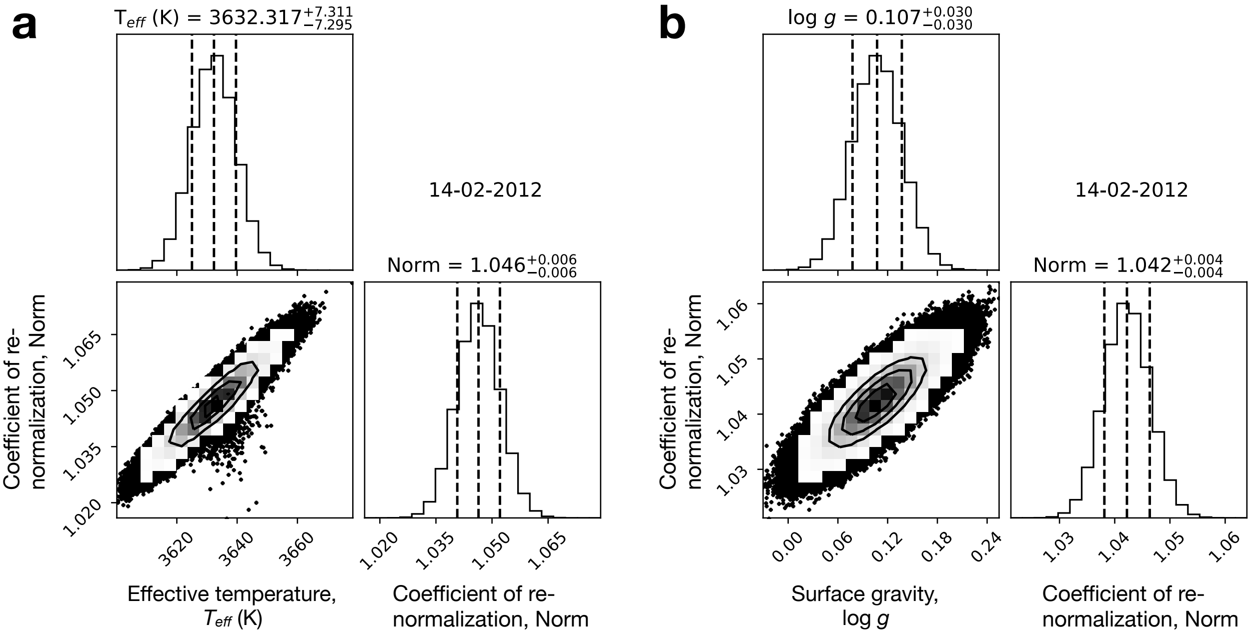

Supplementary Figure 2: **Corner plots with the final effective temperature ($T_{\rm eff}$) and surface gravity (log g) of Betelgeuse obtained on 14-02-2012. a** The corner panels for $T_{\rm eff}$ corresponding to the fit of the region at 7700 − 7900 Å shown in **Figure 7e**. **b** The corner panels for log g corresponding to the fit of the wings of Ca II 8542 Å and 8662 Å lines shown in **Supplementary Figure 4a**. On each panel the diagonal shows the marginalized posteriors. The subsequent covariances between all the parameters are in the corresponding 2D histograms. The vertical dashed lines represent the 16, 50 and 84 percentiles. The best-fit parameters for $T_{\rm eff}$ and log g are presented on the top of each panel. Uncertainty for each parameter ($T_{\rm eff}$ and log g) is defined as the difference between the 84th and 50th percentile as the upper limit, and the difference between the 50th and 16th percentile as the lower limit.The data of observations are given in dd-mm-yyyy format.

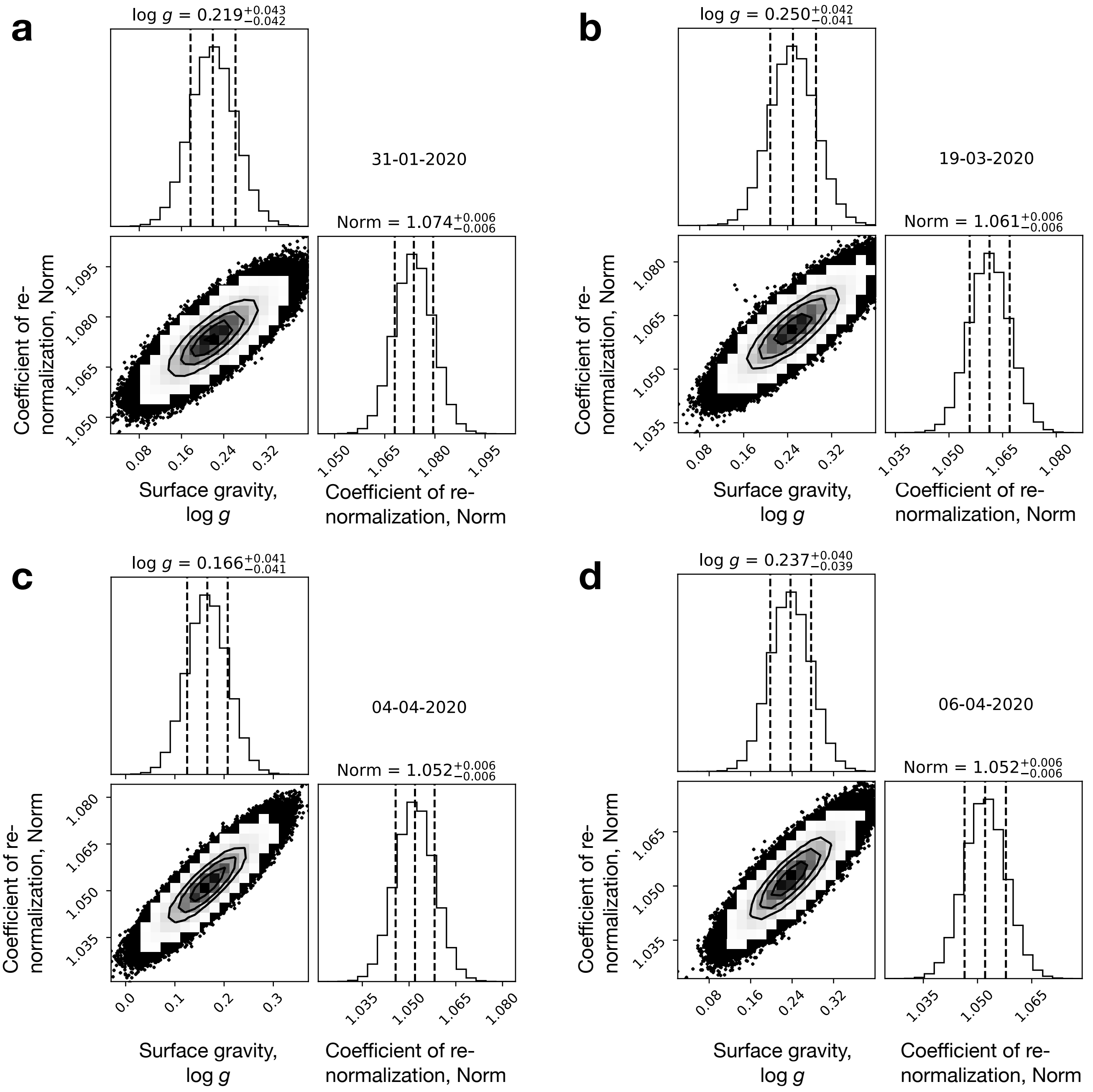

Supplementary Figure 3: **Four corner plots with the final surface gravity (log g) of Betelgeuse obtained at four epochs 31-01-2020, 19-03-2020, 04-04-2020, and 06-04-2020.** **a** The best-fit log g and its uncertainties obtained on 31-01-2020. The corner panels for log g corresponding to the fit of the wings of Ca II 8542 Å and 8662 Å lines shown in **Supplementary Figure 4b**. **b** The best-fit log $g$and its uncertainties obtained on 19-03-2020. **c** The best-fit log g and its uncertainties obtained on 04-04-2020. **d** The best-fit log g and its uncertainties obtained on 06-04-2020. On each panel, the diagonal shows the marginalized posteriors. The subsequent covariances between all the parameters are in the corresponding 2D histograms. The vertical lines represent the 16, 50 and 84 percentiles. The best-fit parameters for log g and their uncertainties are presented on the top of each panel. Uncertainty for log g is defined as the difference between the 84th and 50th percentile as the upper limit, and the difference between the 50th and 16th percentile as the lower limit. The data of observations are marked in dd-mm-yyyy format.

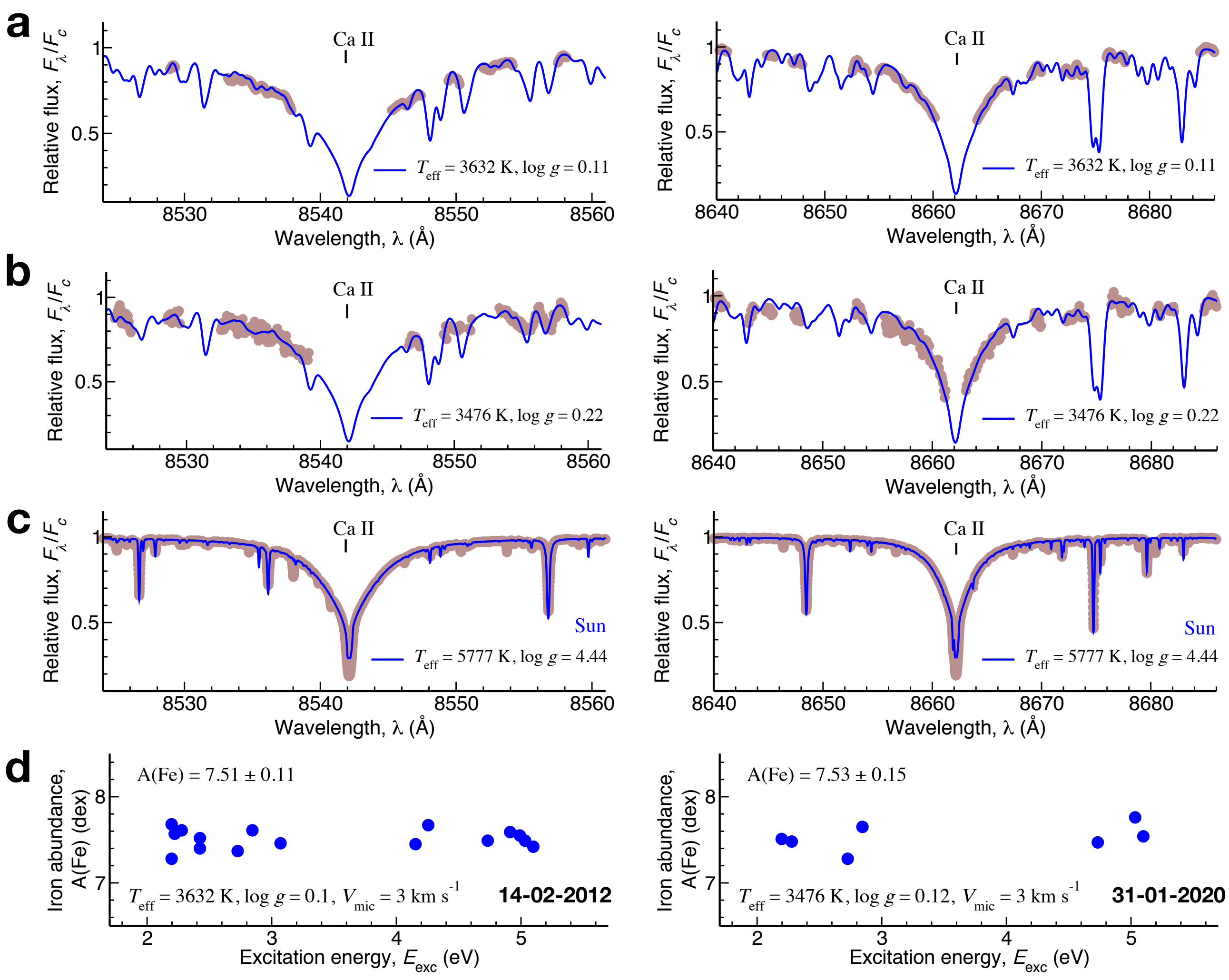

Supplementary Figure 4: **Best fits of the wings of Ca II lines at 8542 Å and 8662 Å.** **a** Best fits of the wings of Ca II lines at 8542 Å and 8662 Å obtained on 14-02-2012. **b** Best fits of the wings of Ca II lines at 8542 Å and 8662 Å obtained on 31-01-2020. **c** Best fits of the wings of Ca II lines at 8542 Å and 8662 Å of the Sun, which was used as benchmark star in this study. The central core of each Ca II lines is marked on the panels **a**, **b**, and **c**. The theoretical profiles (blue solid curves) are calculated with parameters, effective temperature ($T_{\rm eff}$), surface gravity (log g), microturbulent velocity ($V_{\rm mic}$ = 3 km s^{-1}) and convolved with the instrumental profile according to the spectral resolution presented in **Supplementary Table 3**. Relative flux is calculated as a ration of the flux at particular wavelength (F_λ) to the flux in continuum (F_c). **d** The iron abundances (A(Fe)) vs. the excitation energy ($E_{\rm exc}$) of the lower level for investigated Fe I lines for two epochs of observations: 14-02-2012 and 31-01-2020. The parameters ($T_{\rm eff}$, log g and $V_{\rm mic}$) and mean values of the iron abundances with standard deviation are presented. Source data are provided as a Source Data file.

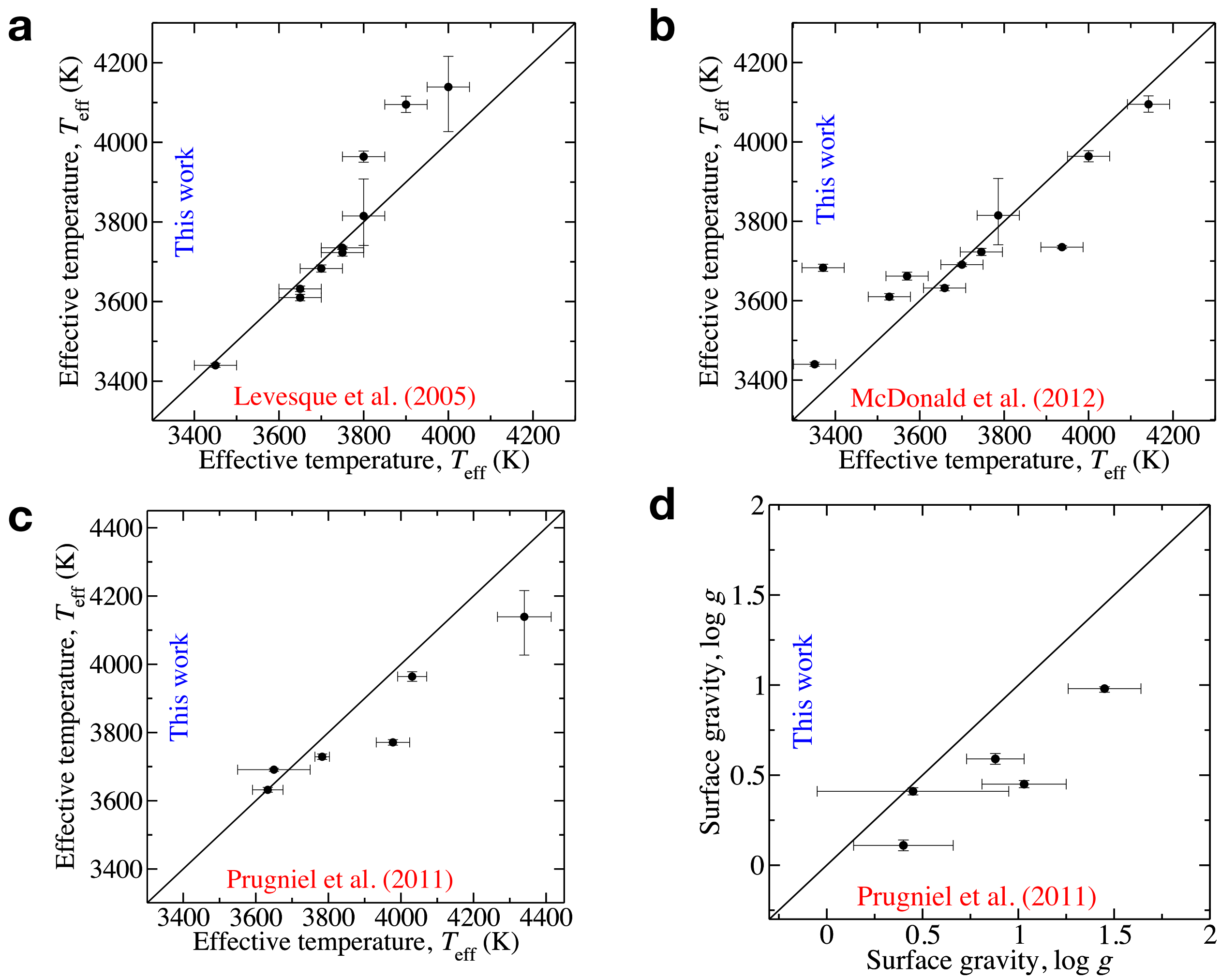

Supplementary Figure 5: **The comparison of effective temperatures ($T_{\rm eff}$) and surface gravities (log g) obtained in this study with previous studies.** **a** The comparison of $T_{\rm eff}$ obtained in this study with supp. ref. 2. The uncertainty of $T_{\rm eff}$ in Levesque et al. (2005) is adopted to be ΔT = 50 K. **b** The comparison of $T_{\rm eff}$ obtained in this study with supp. ref. 3. The uncertainty of $T_{\rm eff}$ in McDonald et al. (2012) is adopted to be ΔT = 50 K. **c** The comparison of $T_{\rm eff}$ obtained in this study with supp. ref. 4. The uncertainty of $T_{\rm eff}$ in Prugniel et al. (2011) is taken from supp. ref. 4. **d** The comparison of log g obtained in this study with supp. ref. 4. The uncertainty of log g in Prugniel et al. (2011) is taken from supp. ref. 4. The uncertainty of $T_{\rm eff}$ and log g in this work is taken as the difference between the 84th and 50th percentile as the upper limit, and the difference between the 50th and 16th percentile as the lower limit. Source data are provided as a Source Data file.

Supplementary Table 1: **CN molecular lines.** Wavelength (λ), excitation energy ($E_{\rm exc}$), and oscillator strength ($\log gf$) for each CN molecular line investigated in this study are presented. Source data are provided as a Source Data file.

λ (Å)	$E_{\rm exc}$ (eV)	$\log gf$
10179.849	0.961	-1.444
10179.734	1.243	-0.863
10179.831	1.177	-1.076
10223.483	1.012	-1.433
10367.098	1.148	-1.394
10376.171	1.148	-1.389
10377.313	1.388	-1.029

Supplementary Table 2: **Chemical abundances in Betelgeuse.** The chemical abundances of C, N, O, Na, Mg, Ca, Ti, Cr, Fe, and Sr are presented under the scenario of variable temperature for five epochs of Betelgeuse's observations 14-02-2012, 31-01-2020, 19-03-2020, 04-04-2020, 06-04-2020. Solar chemical composition are calculated with the same line list and presented for reference. Uncertainties are calculated as standard deviation. Source data are provided as a Source Data file.

Element	14-02-2012	31-01-2020	19-03-2020	04-04-2020	06-04-2020	Sun
C	8.43 ± 0.03	8.40 ± 0.05	8.41 ± 0.03	8.41 ± 0.05	8.41 ± 0.05	8.43 ± 0.03
N	8.60 ± 0.03	8.60 ± 0.05	8.60 ± 0.03	8.60 ± 0.05	8.60 ± 0.05	7.87 ± 0.03
O	8.83 ± 0.03	8.83 ± 0.05	8.80 ± 0.03	8.81 ± 0.05	8.81 ± 0.05	8.69 ± 0.03
Na	6.71	6.77	6.70	6.77	6.77	6.14
Mg	7.68 ± 0.05	7.69 ± 0.01	7.73 ± 0.02	7.73 ± 0.07	7.75 ± 0.01	7.58 ± 0.07
Ca	6.34	6.34	6.34	6.34	6.34	6.37
Ti	5.09	5.11 ± 0.16	5.18 ± 0.18	5.32 ± 0.22	5.21 ± 0.23	4.95
Cr	5.42 ± 0.11	5.46 ± 0.17	5.42 ± 0.17	5.47 ± 0.12	5.32 ± 0.04	5.60 ± 0.13
Fe	7.51 ± 0.11	7.53 ± 0.15	7.47 ± 0.09	7.53 ± 0.12	7.48 ± 0.16	7.41 ± 0.09
Sr	4.62 ± 0.06	4.63 ± 0.09	4.64 ± 0.08	4.72 ± 0.04	4.64 ± 0.11	3.30 ± 0.07

Supplementary Table 3: **Observation list of Betelgeuse.** Julian date (JD - 2450000), mean visual magnitude (V), exposure time (t_{exp}), spectral resolution (R), and signal-to-noise ratio (S/N) are ptresented. Date of observations are presented in dd-mm-yyyy format. Uncertainties for V are calculated as standard deviation. Source data are provided as a Source Data file.

Date	JD - 2450000 (d)	V (mag)	Telescope / Spectrograph	Spectral range (Å)	t_{exp} (s)	R	S/N
06-04-2020	8945.6	0.691 ± 0.102	1 m WO / WES	3710 - 11 000	480	40 000	80
04-04-2020	8943.6	0.754 ± 0.125	1 m WO / WES	3710 - 11 000	480	40 000	100
19-03-2020	8927.6	1.144 ± 0.126	1 m WO / WES	3710 - 11 000	1200	40 000	500
31-01-2020	8879.6	1.609 ± 0.137	1 m WO / WES	3710 - 11 000	1200	40 000	100
14-02-2012	6702.6	0.426 ± 0.296	3.6 m CFHT / ESPaDOnS	3690 - 10 480	0.8	65 000	800

Supplementary Table 4: **Atomic data of Fe I lines.** Wavelength (λ), excitation energy (E_{exc}), oscillator strength (log gf), and van der Waals broadening constant (log γ_6)) for each Fe I line are presented. Source data are provided as a Source Data file.

λ (Å)	E_{exc} (eV)	log gf	log γ_6
8239.127	2.424	-3.18	-7.82
8945.189	5.033	-0.22	-7.53
8946.260	2.845	-3.509	-7.79
8950.188	4.154	-2.425	-7.54
9012.075	4.991	-0.311	-7.53
9013.977	2.278	-3.839	-7.79
9019.744	5.099	-0.988	-7.53
9084.184	4.256	-2.24	-7.54
9100.446	4.913	-1.158	-7.54
10081.393	2.424	-4.537	-7.79
10167.466	2.197	-4.117	-7.80
10195.105	2.727	-3.58	-7.81
10216.313	4.733	-0.063	-7.54
10218.408	3.071	-2.76	-7.65
10265.217	2.222	-4.537	-7.80
10340.885	2.197	-3.577	-7.80

Supplementary Table 5: **Iron abundances in Betelgeuse and the Sun.** Iron abundances are obtained from Fe I lines with wavelengths (λ) under the scenario of variable temperature for five epochs of observations 14-02-2012, 31-01-2020, 19-03-2020, 04-04-2020, 06-04-2020. Solar iron abundances are calculated with the same line list and presented for reference. Source data are provided as a Source Data file.

λ (Å)	14-02-2012	31-01-2020	19-03-2020	04-04-2020	06-04-2020	Sun
8239.127	7.40	–	7.46	7.60	–	7.31
8945.189	7.49	7.76	–	–	–	7.31
8946.260	7.61	7.65	7.46	7.59	7.57	–
8950.188	7.45	–	–	–	–	–
9012.075	7.55	–	7.46	7.63	–	–
9013.977	7.61	7.48	–	7.40	–	7.47
9019.744	7.42	7.54	–	–	–	7.30
9084.184	7.67	–	–	–	–	7.37
9100.446	7.59	–	7.39	7.67	7.56	–
10081.393	7.52	–	–	–	–	7.57
10167.466	7.68	–	7.42	–	–	7.44
10195.105	7.37	7.28	7.64	7.59	7.21	7.50
10216.313	7.49	7.47	–	7.35	7.46	7.44
10218.408	7.46	–	–	7.44	7.59	7.36
10265.217	7.57	–	–	–	–	7.47
10340.885	7.28	7.51	–	–	–	7.38

Supplementary Table 6: **Characteristics of observed spectra of red supergiants.** Star and its name, spectral type (Sp.T.), visual magnitude from SIMBAD data base (V), date of observations in dd-mm-yyyy format (Obs. Date), telescopes (Canada France Hawaii Telescope (CFHT) and Very Large Telescope 2 (VLT2)), spectral resolution (R), and signal-to-noise ratio (S/N)) are presented. Source data are provided as a Source Data file.

Star	Name	Sp.T.	V (mag)	Obs. Date	Telescope	Spectral range (Å)	R	S/N
HD 36389	119 Tau	M2 I	4.33	19-10-2010	3.6 m CFHT	3690 - 10 480	65 000	400
HD 44537	psi01 Aur	M0 I	4.75	17-11-2010	3.6 m CFHT	3690 - 10 480	65 000	400
HD 49331	–	M1 Ib-IIa	5.06	17-02-2003	8 m VLT2	3300 - 10 400	80 000	1500
HD 50877	omi01 CMa	K2.5 I	3.87	03-11-2014	3.6 m CFHT	3690 - 10 480	65 000	400
HD 52877	σ CMa	M1.5 I	3.47	03-11-2014	3.6 m CFHT	3690 - 10 480	65 000	400
HD 146051	δ Oph	M0.5 III	2.75	14-07-2011	3.6 m CFHT	3690 - 10 480	65 000	400
HD 156014	α Her	M5 I	3.06	03-03-2010	3.6 m CFHT	3690 - 10 480	65 000	400
HD 165674	VX Sgr	M8.5 Ia	6.52	15-08-2006	3.6 m CFHT	3690 - 10 480	65 000	300
HD 192909	32 Cyg	K7 Ib-II	3.98	08-12-2009	3.6 m CFHT	3690 - 10 480	65 000	400
HD 200905	ξ Cyg	K4.5 I	3.73	09-06-2011	3.6 m CFHT	3690 - 10 480	65 000	400
HD 206936	μ Cep	M1 I	4.08	09-12-2006	3.6 m CFHT	3690 - 10 480	65 000	400
HD 208816	VV Cep	M2 Ia-Iab	4.9	27-07-2010	3.6 m CFHT	3690 - 10 480	65 000	400
HD 210745	ζ Cep	K1.5 I	3.35	14-07-2011	3.6 m CFHT	3690 - 10 480	65 000	400
HD 216946	V424 Lac	M0 I	4.94	16-11-2010	3.6 m CFHT	3690 - 10 480	65 000	400
HD 217906	β Peg	M2.5 II-III	2.42	08-06-2011	3.6 m CFHT	3690 - 10 480	65 000	400

Supplementary References

1. Harper, G. M., Guinan, E. F., Wasatonic, R. and Ryde, N. The Photospheric Temperatures of Betelgeuse during the Great Dimming of 2019/2020: No New Dust Required. *Astrophysical Journal* **905**, 34 (2020).

2. Levesque, Emily M., et al. The Effective Temperature Scale of Galactic Red Supergiants: Cool, but Not As Cool As We Thought. *Astrophysical Journal* **628**, 973 (2005).

3. McDonald, I., Zijlstra, A. A., and Boyer, M. L. Fundamental parameters and infrared excesses of Hipparcos stars. *Monthly Notices of the RAS* **427**, 343 (2012).

4. Prugniel, Ph., Vauglin, I., and Koleva, M. The atmospheric parameters and spectral interpolator for the MILES stars *Astronomy and Astrophysics* **531**, A165 (2011).
